# The Sphingolipid-Signaling Pathway as a Modulator of Infection by SARS-CoV-2

**DOI:** 10.3390/cimb45100503

**Published:** 2023-09-28

**Authors:** Simona Fenizia, Melania Gaggini, Cristina Vassalle

**Affiliations:** 1 Istituto di Fisiologia Clinica, Italian National Research Council, Via Moruzzi 1, 56124 Pisa, Italy; 2 Fondazione CNR-Regione Toscana G. Monasterio, Via Moruzzi 1, 56124 Pisa, Italy

**Keywords:** viral infection, COVID-19, biomarkers, lipids, ceramides, sphingosine-1-phosphate

## Abstract

Ceramides and other related sphingolipids, important cellular components linked to metabolic homeostasis and cardiometabolic diseases, have been found to be involved in different steps of the SARS-CoV-2 life cycle. Hence, changes in their physiological levels are identified as predictors of COVID-19 severity and prognosis, as well as potential therapeutic targets. In this review, an overview of the SARS-CoV-2 life cycle is given, followed by a description of the sphingolipid metabolism and its role in viral infection, with a particular focus on those steps required to finalize the viral life cycle. Furthermore, the use and development of pharmaceutical strategies to target sphingolipids to prevent and treat severe and long-term symptoms of infectious diseases, particularly COVID-19, are reviewed herein. Finally, research perspectives and current challenges in this research field are highlighted. Although many aspects of sphingolipid metabolism are not fully known, this review aims to highlight how the discovery and use of molecules targeting sphingolipids with reliable and selective properties may offer new therapeutic alternatives to infectious and other diseases, including COVID-19.

## 1. Introduction

**Figure 1:**
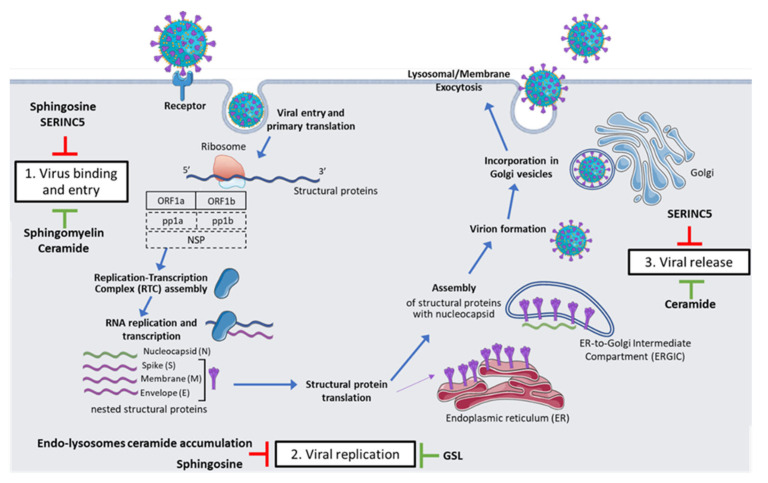
The life cycle of SARS-CoV-2 and the different levels at which sphingolipids can act. Figure generated using PowerPoint. Templates for viruses, receptors, and cellular components are under the Creative Common License (CC-BY-4.0) and have been adapted and modified. Green signs: induction; red signs: inhibition.

The COVID-19 pandemic, caused by severe acute respiratory syndrome coronavirus 2 (SARS-CoV-2), has represented a public health emergency of international concern, spreading rapidly from its onset in China at the end of 2019 and often overwhelming the global healthcare systems [1]. In this context, sphingolipids (SLs), major components of cell membrane architecture, were recently shown to modulate SARS-CoV-2 infection. In particular, ceramides (Cers), a group of bioactive lipids belonging to the SL family, contain sphingoid bases as the backbone of their molecular structure; depending on their specific characteristics (e.g., fatty acyl chain length and subcellular location), these molecules play a variety of regulatory functions in important physiological pathways [2]. Cers and other related SLs have been found to be involved in different steps of the viral life cycle, including the uptake of SARS-CoV-2 (likely through acid sphingomyelinase (ASM) activation, whose inhibition reduces SARS-CoV-2 entry into the cells), viral gene expression and replication, virion release, apoptosis, and viral immune modulation and escape [3,4,5] (Figure 1). Other findings highlight the role of the SL sphingosine-1-phosphate (S1P) as a relevant predictor of the severity of COVID-19 (intensive care unit admission) as well as the patient’s prognosis [6,7]. Moreover, the activation of sphingosine-1-phosphate receptor 1 (S1PR1) triggers the inflammatory response related to SARS-CoV-2 by stimulating interferon alpha (INF-α), which may inhibit viral infection and decrease viral load [8]. In this review, an overview of the SARS-CoV-2 life cycle and a description of the SL metabolism are discussed in order to highlight the interplay between the virus and SLs. Indeed, the ability of the virus to actively affect lipid composition is a key step to satisfactorily finalizing the viral life cycle. Additionally, we report available data on the modifications of the SL profile in patients with COVID-19 and their relationship with the severity of the disease and the onset of clinical complications. Taken together, these results strongly suggest that SLs exert a critical, although still undervalued, role in controlling the viral life cycle and the immune response; thus, this review also aims to discuss the possibility of targeting SL metabolism to face the viral infection. This research area is still in its early stages, and many aspects of the interaction between SLs and viruses are still enigmatic. However, because several SL metabolism inhibitors are already in use in a variety of clinical contexts, their application in the viral infection scenario (including COVID-19) looks promising, although further research in future trials is required. Hence, in the last part of this review article, research perspectives and current challenges in this research field are discussed. Despite the complexity of the SL metabolism, with many aspects yet to be elucidated, the discovery and application of pharmacological tools that can reliably and selectively modulate SLs may provide new supplementary therapeutic options in the near future, promising to address many diseases, including viral infections.

## 2. SARS-CoV-2 Life Cycle

SARS-CoV-2 is a positive, single-stranded RNA virus whose 3′-terminal genomic sequence contains two large open reading frames, 1a and 1b (ORF1a and ORF1b), encoding several structural proteins, which consist of spike (S), envelope (E), membrane (M), and nucleocapsid (N) proteins [9]. While the RNA genome is encapsidated by N, the proteins M and E are responsible, during the assembly process, for its incorporation into the viral particle; S trimers, instead, protrude from the host-derived viral envelope and provide specificity for cellular entry receptors [10]. The SARS-CoV-2 virus mainly infects mucus-producing goblet cells and ciliated cells in the respiratory system, although infection of lung endothelial cells has also been observed [9].

The life cycle of this virus (Figure 1) is characterized by three main distinctive steps: (1) virus binding and entry; (2) viral replication; and (3) viral release.

### 2.1. Virus Binding and Entry

As shown in Figure 1, the infection starts with the binding of the SAR-CoV-2 S protein to cellular receptors, such as angiotensin-converting enzyme 2 (ACE2). Thus, both the expression as well as the tissue location of entry receptors directly influence viral tropism and pathogenicity [10]. Coronavirus S proteins are class I homotrimeric fusion glycoproteins, which were placed in multiple sequences into the virion membrane so that the virion assumes a crown-like appearance [11,12]. These proteins consist of two functionally distinct subunits, S1 and S2, cleaved by proprotein convertases (like furin) within the virus-producing cell. While the S1 subunit binds ACE2 with the receptor-binding domain (RDB), the S2 anchors the S protein to the membrane and expresses the cellular components needed to mediate membrane fusion upon cellular infection [10,12]. Cleavage of coronavirus S proteins is thus required for fusion, while cell entry relies on the target-cell proteases TMPRSS2 (expressed on the cell surface, it activates the S protein at the plasma membrane level) and cathepsin L, which determine the S protein activation within the endolysosome [10,12]. Following receptor contact, conformational changes occur, triggering the steps that lead to the fusion of the virus with the cellular membrane and the penetration of the viral ribonucleoprotein complex into the cytoplasm [9].

### 2.2. Viral Replication

Upon entrance, the incoming genomic RNA is released and uncoated; subsequently, at the 5′-terminal sequence of the genome, ORF1a and ORF1b are translated to pp1a and pp1b polyproteins, respectively. These newly formed polyproteins are, in turn, proteolytically cleaved to non-structural proteins (NSPs); in particular, pp1a cleavage leads to the formation of NSPs from 1 to 11 (NSP1–11), while pp1b is cleaved to form NSPs from NSP12 to NSP16. Combined together, NSPs form the replication-transcription complex (RTC), which includes the RNA-dependent RNA polymerase (RdRp) and drives viral replication and subgenomic mRNA transcription. The newly formed mRNAs are then further translated into the structural proteins S, E, and M, which enter endoplasmic reticulum (ER) membranes, while the N protein forms a nucleocapsid together with RNA. Both the nucleocapsid and the other structural proteins will leave the ER to reach the ER-to-Golgi intermediate compartment (ERGIC) [9,13]. During its life cycle, SARS-CoV2 expresses typical perinuclear double-membrane vesicles, as well as convoluted membranes and small open double-membrane spherules; all these structures form an ideal setting for viral genomic RNA replication and transcription, generating full-length RNA copies to integrate into newly formed viral particles [10].

### 2.3. Viral Release

Once in the ERGIC, interactions between the structural proteins and the novel N-encapsidated genomic RNA drive the budding of secretory vesicles, which lead to the virion excretion, by lysosomal or membrane exocytosis, from the infected cell and the subsequent infection of other host cells [10,14]. In the span of multiple rounds of replication, each single virus that manages to enter a host cell can form millions of virions, which in turn can induce the infection, thus triggering a continuous loop that ends only when it can be inhibited and overcome [15].

## 3. Sphingolipids: Metabolism and Function

**Figure 2:**
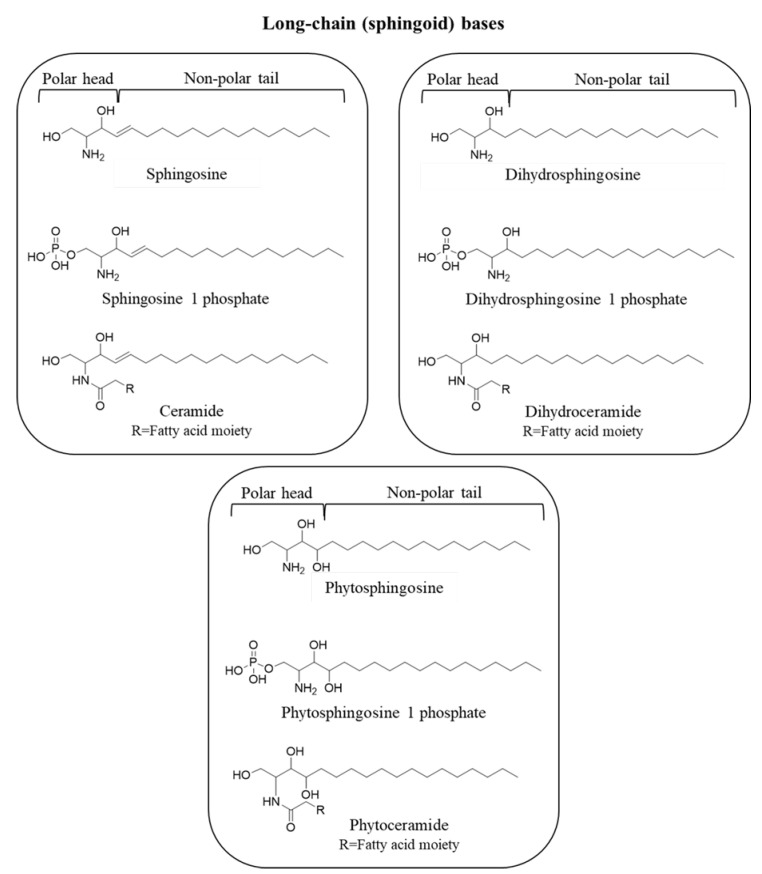
Sphingoid bases and correspondent sphingolipids. CambridgeSoft ChemDraw Ultra 12.0 was used to draw the chemical structures.

As previously stated, SLs are a major class of membrane lipids that play an essential structural role in cell membranes and also act as signaling molecules and modulators of key metabolic pathways, such as apoptosis, cell proliferation, enzymatic activities, inflammation, and viral infections [16,17,18,19]. Mammalian SLs are amphipathic molecules whose hydrophilic region contains phosphate groups, sugar residues, and/or hydroxyl groups, while the hydrophobic region is characterized by sphingoid bases (or long-chain bases) as the defining components, which in living tissues are usually linked to a fatty acid via an amide bond [20]. Sphingoid bases consist of eighteen carbon amino-alcohol backbones (2-amino-1,3-dihydroxy-alkanes) and, in some cases, a distinctive *trans*-double bond in position 4 (Figure 2) [21]. Free sphingoid bases are bioactive components: thanks to their amphipathic nature, they can indeed spread and flip between membrane leaflets, where interaction with specific receptors occurs [22]. Long-chain bases include the following: sphingosine (Sph), a C-18 aliphatic amine with an amine group at C2, two hydroxyl groups at C1 and C3, and a double bond in *trans* configuration at C4; phytosphingosine, characterized by a saturated aliphatic chain and an additional hydroxyl group at C4; and sphinganine (dihydrosphingosine), which lacks the double bond (Figure 2) [23]. Structural modifications in these basic structures, particularly in the alkyl chain length, serve as backbones to achieve further molecular complexity and give rise to the vast family of SLs [22]. Hence, phosphorylation of the C1 hydroxyl group yields S1P, phytosphingosine-1-phosphate, and dihydrosphingosine-1-phosphate, which are important signaling molecules; if the long chain moiety reacts with fatty acid molecules through a ceramide synthase (CerS), correspondent Cer, phytoceramide, or dihydroceramide (DHCer) are formed (Figure 2) [22].

**Figure 3:**
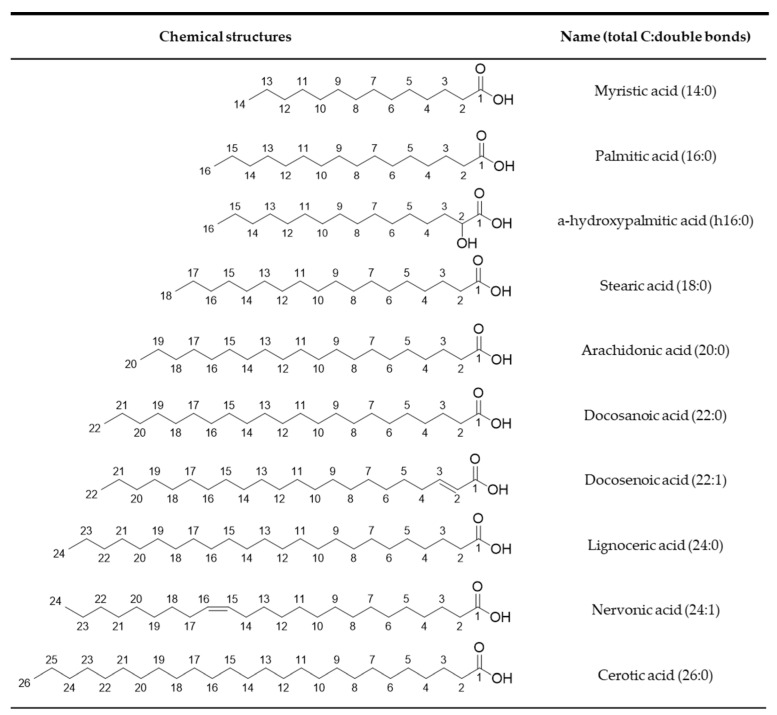
Major fatty acid moieties building up sphingolipids. CambridgeSoft ChemDraw Ultra 12.0 was used to draw the chemical structures.

Fatty acids that build up Cers are generally mono-unsaturated or saturated, with chain lengths ranging from 14 to 26 carbons, in some cases even more (Figure 3) [24]. Because of the high variety of fatty acid moieties, Cers represent an entire class of metabolites, each playing a specific biological function depending on the acyl group that becomes attached [22].

The biosynthesis of simpler SLs serves to form both precursors and breakdown products for the more complex ones. In this context, glycosphingolipids (GSLs) are a group of highly complex lipids consisting of several SL species that differ in the order and type of sugar residues attached to their headgroups. Next to GSsL, sphingomyelins (SMs) are an even more abundant species of complex SLs, characterized by a phosphocholine head group rather than sugar residues. As Cers are the direct precursors of GSLs and SMs, their acyl chain composition is highly diverse, thereby increasing the level of structural variability and abundance according to the different acyl chains that become attached to their C-2 amino groups [22].

Cers, and therefore SLs, are synthesized by three pathways: the de novo pathway, the sphingomyelin pathway, and the salvage/recycling pathway, which individually or coordinately contribute to ceramide synthesis and subsequent cellular responses [25,26].

### 3.1. De Novo Pathway and Ceramide Transport to the Golgi

**Figure 4:**
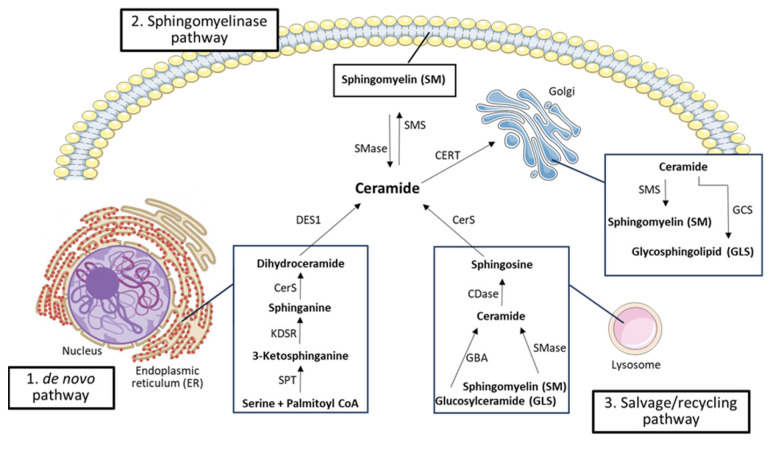
Main pathways of the sphingolipids’ metabolism: de novo, SMase, and salvage/recycling pathways. Figure generated using PowerPoint. Templates for cellular components are under the Creative Common License (CC-BY-4.0) and have been adapted and modified.

The de novo synthesis pathway (Figure 4) takes place at the cytosolic leaflet of the ER and possibly in ER-associated membranes, such as the perinuclear membrane and mitochondria-associated membranes [27]. Here, a serine palmitoyltransferase (SPT), a rate-limiting enzyme, initiates the condensation of serine and palmitoyl-CoA to produce 3-keto-dihydrosphingosine (3-ketosphinganine), which is reduced to dihydrosphingosine (sphinganine) by a 3-keto-dihydrosphingosine reductase; sphinganine is thus acetylated to DHCer, a single membrane-bound lipid, by a (dihydro) CerS [22,25,27,28]. Six isoforms of CerS have been identified, which catalyze the same chemical reaction but are each specialized in the synthesis of Cer with a different acyl-CoA chain length [29,30]. Once produced, DHCer is acted on by dihydroceramide desaturase-1 (DES1), which introduces a double bond to form the corresponding Cer [28,29]. 

As the newly synthesized Cer is a membrane-bound molecule with low solubility in an aqueous environment, it is transferred from the ER to the Golgi apparatus through two main transport systems [19,22]. The first and most characterized transport mechanism is the non-vesicular Cer transport protein CERT, which specifically delivers Cer (while showing lower affinity towards other SL) from the ER to the Golgi to trigger the synthesis of SMs [27,31]. The second mechanism, on the other hand, is a vesicular transport, which is more specific for long-chain Cer (>C20) [27]: in this case, the specific transporter depends on phosphoinositide-3-kinase activity, which, unlike CERT, has not yet been well characterized [22,29]. Once they reach the Golgi apparatus, Cers are metabolized to form SMs and more complex GSLs [17,22]. 

SMs are major components of cell membranes, synthesized by sphingomyelin synthase (SMS), an enzyme that transfers a phosphorylcholine head group from phosphatidylcholine to Cer, yielding diacylglycerol (DAG) and SM, bioactive lipids with different effects on cell growth and survival [19,22]. The enzyme SMS is made up of six transmembrane domains with catalytic sites oriented toward either the Golgi lumen or extracellular space [22]. Moreover, two SMS isoforms have been identified (SMS1 and SMS2), both present in the Golgi apparatus, but SMS2 is expressed in the plasma membrane as well, so it is also responsible for keeping the balance of the SM content at the level of the plasma membrane [22,29,32]. In addition to SM, Cer can be glycosylated to form GSL; among them, glucosylceramide (in the Golgi) and galactosylceramide (in the ER) play significant roles in cell regulation and myelin structure and function, respectively. Moreover, through a ceramide kinase (CerK), Cer can also drive the formation of ceramide-1-phosphate (C1P), which regulates cell growth and inflammation and serves as a docking site for phospholipase A2, thereby boosting arachidonic acid production (Figure 4) [29].

### 3.2. Sphingomyelinase Pathway

In addition to the de novo pathway, Cer can also be produced through SM hydrolysis catalyzed by sphingomyelinases (SMases), enzymes that cleave SMs to generate free phosphocholine and Cer (Figure 4). Based on their pH range, SMases are usually classified into acid, alkaline, and neutral SMases, which are differently expressed in the various tissues. Alkaline SMases are more abundant in the liver and intestine, where they regulate the digestion of dietary SM; on the other hand, acid and neutral SMases are widely expressed and mainly coordinate SM catabolism in several tissues [22]. The hydrolysis of SMs by SMases has emerged as a significant route of stress-induced Cer production; conversely, the enzyme SMS catalyzes the transfer of the headgroup of phosphatidylcholine to Cer, leading to the formation of SMs and DAG [27]. While this mechanism has been proposed to modulate SM, DAG, and Cer levels and be involved in NFkB activation, the breakdown of SMs through acid SMases is a significant route in the production of Cer and other lipids, such as S1P, the last sphingolipid preceding the final degradation of SLs. After the conversion of SM to Cer by acid SMases, ceramidases (CDases) deacylate Cer and produce Sph, upon removal of a fatty acid from the amide bond [27].

Similar to SMases, CDases are classified into acid, neutral, and alkaline according to their optimal pH and their localization: whereas the acid CDase (ASAH1) is mainly found in lysosomes and is involved in medium-chain Cer deacylation, the neutral (ASAH2) regulates Sph and S1P production at the plasma membrane level. Concerning alkaline CDases (ACER), three forms are available: ACER1, mainly expressed in the ER in epidermal cells; ACER2, in the Golgi apparatus and placenta; and ACER3, localized in both the Golgi and ER and also widespread among different tissues compared with the other two alkaline CDases [29]. Once produced, Sph passes through the lysosome and is either re-acylated to Cer by CerS or phosphorylated by sphingosine kinases (SphK) to produce S1P. The enzyme SphK has two isoforms, namely SphK1 and SphK2, which catalyze the same process but in different regions of the cell [28]. SphK1 is mainly a cytosolic enzyme whose residues bind acidic phospholipids, aiding in SphK1 intracellular localization; SphK2, on the other hand, occurs in several cell compartments, including the nucleus, ER, and mitochondria. Although the processes involved in SphK2 regulation are largely unknown, many functional roles of SphK2 are emerging in the modulation of key cellular processes (e.g., apoptosis, cell senescence, aging, and inflammation) [33]. S1P is involved in several physiological processes and plays a role in the regulation of cell survival and proliferation (which are opposite effects to Cer), while SphK1 has been identified as a key signaling enzyme that controls the ratio between pro-survival and pro-apoptotic SL metabolites, a balance known as the “sphingolipid rheostat” [28,29]. S1P degradation enzymes include lipid phosphate phosphohydrolases, which hydrolyze S1P to Sph, and S1P-lyase, which breaks S1P down into hexadecenal and phosphoethanolamine [28,29]. It is noteworthy that there is no complete agreement on whether Smase deserves a pathway for the convertion of SM to Cer: indeed, Smases generate Cer by converting an existing SL to another sphingolipid (Cer). Moreover, although a first description of the Smase pathway, reported by Hannun and colleagues, was included in the context of ceramide generation [26], in several other works it is reported as an independent metabolic path [34,35,36,37,38]. Thus, this topic clearly requires further studies and exploration.

### 3.3. Salvage/Recycling Pathway

The SL salvage pathway (Figure 4) includes all the processes involved in the formation of Cer and those resulting from the catabolism of complex SLs (e.g., SMs and GSLs), which are cleaved into Sph that is subsequently reused to produce Cer via reacylation [26,27]. This pathway accounts for 50–90% of SL biosynthesis and leads to the regeneration of SLs from sphingoid bases. It takes place in acidic subcellular compartments, like endosomes and lysosomes, and involves several enzymes, mainly CDases, SMases, hydrolases, and CerS, to break down SL and GSL [29]. Among hydrolases, the enzyme β-glucosidase acid acts on the β-glycosidic bonds of GSL, hydrolyzing their terminal β-D-glucose residues. Hence, monosaccharide units are released, and Cers are formed. The SMases, instead, convert SMs located in the lysosomes into Cers, which, in turn, are hydrolyzed by CDase, yielding Sph and free fatty acids. These newly formed compounds are able to leave the lysosomes, unlike the Cer molecule; once outside the lysosome, Sph and free fatty acids take part in the Cer biosynthesis pathway, leading to the formation of either new Cers, via CerS, or S1P, via a phosphorylation reaction catalyzed by SphK1-2. As in the sphingomyelinase pathway, S1P is then further metabolized into either Sph or hexadecenal and phosphoethanolamine [27,29].

## 4. Sphingolipids in SARS-CoV-2 Life Cycle

With regards to the class of SLs, among the most studied are S1P and Cer, not only for their role in the association with human diseases, particularly in the context of respiratory pathophysiological conditions [39,40], but also because they are emerging as key factors that could influence host-pathogen interactions. In particular, they have been shown to modulate several viral processes (e.g., inflammation and infection), including those related to SARS-CoV-2. In this context, Cer and S1P play opposite roles since Cer mainly controls cell growth arrest, death, and senescence, whereas S1P regulates cell proliferation and survival [41]. Indeed, researchers demonstrated that the infection of cultured epithelial cells with the SARS-CoV-2 S protein activated the ASM, thus catalyzing the conversion of SMs into Cers [42]. Hence, SLs, in particular Cers and their metabolizing enzymes (e.g., SMases), take part in nearly every step of the viral life cycle. 

### 4.1. Viral Entry

Like many other viruses, SARS-CoV-2 modulates the membrane to allow cellular infection; in this context, sphingolipid-enriched microdomains facilitate fusion at the plasma membrane and modulate endocytosis-mediated uptake and virus release. Accordingly, in the early stages of SARS-CoV-2 infection, binding and fusion of the viral S protein with ACE2, a receptor that is preferentially located at the lipid raft level, occur [43]. Therefore, the fusion process of viruses depends on the lipid composition of the host membrane; in fact, several lipids may inhibit or promote the fusion (i.e., lysophospholipids, phosphatidylethanolamine, and cholesterol) [44].

Given the importance of lipids in both the viral life cycle and the impairment of the host immune response, infectious agents can exploit and modulate the host’s lipid assets to their advantage. Indeed, pathway analysis has shown that SL metabolism is the most altered pathway after viral infection [45]. These alterations in lipid metabolism have also been observed after SARS-CoV-2 infection and may therefore be involved in the development of both pro- and antiviral effects, thus playing a role in the disease progression [46]. In this context, Sph has been shown to affect membrane fluidity and lipid composition (raft membrane); additionally, Sph may interfere with ACE2, hindering virus–receptor interaction [47]. In this regard, using epithelial cell cultures infected with pseudo-viral particles containing the SARS-CoV-2 S protein, Edwards et al. [48] showed that Sph can actually interfere with the binding of the SARS-CoV-2 to its receptor ACE2 by binding itself to ACE2, thus inhibiting the viral S protein receptor-binding domain from the interaction with ACE2 [48].

Since Cers are hydrophobic compounds, they tend to spontaneously associate with each other, forming a sort of platform through which the virus can enter [43]. Indeed, the reduction of Cer through the inhibition of ASM protects against SARS-CoV-2 infection (4). Accordingly, in a cellular model (Vero E6), infection with SARS-CoV-2 was associated with an increase in SL levels 3 h thereafter. In particular, levels of 3-ketosphinganine (d16:0, d18:0, d18:1, and d20:0), sphinganine, Sph, sphinganine-1-phosphate (d18:0-P and Sa1P), DHCer, and Cer were significantly increased in SARS-CoV-2-infected cells [49].

### 4.2. Viral Replication

As in the case of Herpes simplex virus (HSV-1) infection, Sph might inhibit viral propagation and protect against severe disease by reducing viral fusion at the lysosomal level [50]. In fact, when macrophages accumulate HSV-1 in multivesicular bodies, Sph-rich intraluminal vesicles capture the virus and prevent cellular infection. SLs also modulate viral replication, as inhibition of the CDases (by treatment with AKS488 or fluoxetine) increases endo-lysosomal Cer, which inhibits SARS-CoV-2 replication in lysosomal compartments [51]. Furthermore, as a critical step for viral replication, a significant rise in GSL was observed early after SARS-CoV-2 infection; treatment with glucosylceramide synthase (GCS) inhibitors reversed this trend [49]. Likewise, key enzymes involved in the glycerophospholipid metabolism pathway (e.g., phosphatidic acid phosphatase 1) have been found to modulate the replication of SARS-CoV-2 [52].

### 4.3. Viral Release

Inhibition of neutral SMases (through GW4869 or RNAi) alters the composition of extracellular vesicles in two different ways: it impairs exosome release while enhancing microvesicle budding on the plasma membrane [53]. In this regard, lipidomic analyses show a specific lipid composition of localized ordered cholesterol and SL-rich lipid nanodomains in the early Golgi, where viral budding occurs [54]. Moreover, incorporation into budding virions of the cellular transmembrane protein Serine Incorporator 5 (SERINC5), involved in sphingolipid and phosphatidylserine biogenesis, reduces virus infectivity, likely by blocking the virus-cell fusion and thus the entry of SARS-CoV-2 virus-cell fusion. The antiviral effect of SERINC5, however, can be suppressed by the SARS-CoV-2 transmembrane protein ORF7a, which can block the incorporation of overexpressed SERINC5 into budding virions [55].

## 5. Role of Sphingolipids in COVID-19 Severity and Complications

COVID-19-associated acute respiratory distress syndrome (COVID-ARDS) has been indicated as the leading cause of death in COVID-19, while acute lung injury has been linked to SL metabolism disruption. Indeed, dysfunction of SL metabolism leads to the accumulation of Cers [56]. The metabolism of SLs also induces the immune response and inflammation by converting Sph to S1P, resulting in the formation of an S1P gradient and a consequent release of lymphocytes from lymphoid organs into the bloodstream [57]. 

Mass spectrometry-based profiling of Cer in COVID-19 patients showed that palmitoyl Cer (C16:0-Cer) increased 3-fold in the plasma of COVID-ARDS subjects compared with healthy individuals; moreover, a massive increase (almost 9-fold) in C16:0-Cer was detected in autopsied lung tissue obtained from individuals who died of severe COVID-19 [58]. Additionally, in both plasma and lungs, the C16:0-Cer/C24:0-Cer ratios were increased and reversed, respectively, which is consistent with an increased risk of vascular injury [58]. The vascular pathogenic role of C16:0-Cer and the protective role of C24:0-Cer have been shown in epidemiological and clinical studies of populations at risk for a major adverse cardiovascular event [59,60,61]. Cers are generated in plasma membranes by activation of ASM, whose expression is 20-fold higher in endothelial cells than in any other mammalian cell [62,63]. Hence, the increase in C16:0-Cer observed in COVID-19 plasma may reflect endothelial cell ASM activation by oxidative stress, which is common in COVID-19-infected patients [62,63]. Furthermore, through a lipidomic analysis performed by tandem mass spectrometry, reduced serum Sph levels have been found to be closely associated with the development of the symptomatic disease compared to asymptomatic forms [64]. When compared to SARS-CoV-2 antibody-negative controls, the majority of asymptomatic subjects (73%) showed higher levels of ASAH1 in serum, which is in line with the higher Sph levels [64].

Moreover, another study, where lipidomic analysis was performed on the plasma of COVID-19 patients and healthy controls, highlighted lower S1P levels in infected subjects compared to controls, with S1P levels that increased again at hospital discharge [65]. Other data obtained from plasma lipidomic analysis from three cohorts (i.e., COVID-19 uninfected subjects, infected with mild symptoms, and infected with severe symptoms) demonstrated that Cer subclasses Cer(d18:0/24:1), Cer(d18:1/24:1), and Cer(d18:1/22:0) increased 48-, 40-, and 33-fold, respectively, in infected plasma samples and to 116-, 91-, and 50-fold, respectively, in plasma samples with respiratory distress [66]. This rise demonstrates the toxic effect that Cer can exert on lung endothelial cells, thus leading to the pathogenesis of several conditions associated with pulmonary vascular dysfunction [67].

## 6. Sphingolipids as Potential Therapeutic Targets in SARS-CoV-2 Infection

A considerable range of pathophysiological conditions related to COVID-19 emerged from the study of SL metabolism, generating a great deal of concern on the one hand and growing scientific interest in the study of modulating this metabolic pathway on the other. In this context, some drugs, such as PCSK9 inhibitors or statins, may decrease SL levels [68,69]. In particular, statins, along with other FDA-approved lipid-lowering drugs such as metformin, hydroxychloroquine, and cyclodextrins, can disrupt SL- and cholesterol-rich lipid zones, where receptors and molecules involved in pathogen recognition and cellular signaling, including those related to SARS-CoV-2 infection, are most highly expressed [70]. Moreover, these drugs reduce the levels of pro-inflammatory molecules (e.g., TNF-α and IL-6) and/or affect the autophagic process involved in the replication and clearance of the virus [71]. Other studies also showed that statins may benefit COVID-19 patients by stabilizing atherosclerotic plaques and preventing coronary events [72], which is a benefit that was also confirmed by two other meta-analyses, where a reduction in intensive care unit (ICU) hospitalization, mortality, and mechanical ventilation requirements was reported as a result of statin treatment [73,74]. However, a more recent meta-analysis, based on seven randomized controlled trials (1830 subjects), did not find a statistically significant difference in all-cause mortality (risk ratio (RR): 0.92, 95% confidence interval (CI): 0.75–1.13), in length of hospital stay (weighted mean difference: −0.21 days, 95% CI: −1.01 to 0.59 days), in ICU admission (RR: 1.84, 95% CI: 0.45–7.55), and in mechanical ventilation (RR: 1.09, 95% CI: 0.70–1.70), raising concerns about the routine use of statins in COVID-19 patients [75]. Another noteworthy concern is that all these results are based on the previous use of statins against infection and cannot be extended or applied to the introduction of statin treatment after the onset of COVID-19 infection, which requires further investigation.

The use of PCSK9 inhibitors for COVID-19 has been considered an opportunity to enhance the antiviral action of interferon in patients with hypercholesterolemia [76]. Actually, PCSK9 inhibition effectively reduced the primary endpoint of death or need for intubation, as well as IL-6 levels in severe COVID-19 compared to placebo; moreover, patients with more severe inflammation at the time of randomization had better survival with PCSK9 inhibition than placebo, indicating that the intensity of inflammation may drive therapeutic benefits [77].

Natural products, such as resveratrol (RSV), a phenolic compound with antioxidant activity, can also modulate SL metabolism [18]. In particular, in addition to its antioxidant and immunomodulatory properties, RSV also inhibits SphK and decreases SMs and S1P, thus reducing downstream effects and increasing Cer levels [18]. RSV seems to act in different phases of the virus life-cycle and through several mechanisms (i.e., its antiviral efficacy is achieved by inhibiting virus entry into cells and viral replication; increasing autophagy while reducing the expression of pro-inflammatory molecules and inhibiting cytokine storm; modulating the immune response; and preventing thrombotic events) [78]. In particular, recent studies have investigated the main RSV-related mechanisms in COVID-19 patients using a pharmacological network approach and bioinformatics gene analysis, showing that RSV may act by reducing the pro-inflammatory signaling pathway involving IL-17, TNF, and NF-κB [79]. Actually, the importance of RSV effects arising from SL modulation has not yet been thoroughly studied, especially the effects of RSV on SL mediators with a key role in inflammation (e.g., Cer, C1P, and S1P), which may further contribute to the role of RSV in COVID-19 [80].

Some of the enzymes involved in the SL pathways, such as CerS, DES, and SMase, which catalyze the final steps of the de novo biosynthesis pathway, the salvage pathway, and SM hydrolysis, respectively, have been identified as attractive targets for therapy, including antiviral drugs. Thus, several molecules affecting SL metabolism have either been tested in in vitro or in vivo animal models, have even already been used in clinical practice in different settings (e.g., multiple sclerosis or neurodegenerative diseases such as Parkinson’s disease and depression), or are being studied, either alone or in addition to traditional therapies, against different types of cancer and to prevent atherosclerosis [36]. Among these, the fungal product Myriocin inhibits the enzyme SPT, which catalyzes the initial step of SL biosynthesis. Its synthetic-derived compound, fingolimod (FTY720), is an S1P analogue that acts mainly through modulation of four out of the five S1P receptors (S1PR1 and S1PR 3–5); it has been approved by the FDA for multiple sclerosis and is currently being studied for cancer applications [81]. Several studies on the use of FTY720 in patients with multiple sclerosis and SARS-CoV-2 infection are available, which suggest that the immunomodulatory effect of S1P analogues appears beneficial in reducing mortality [82]. Conversely, one case report pointed out that the discontinuation of fingolimod during COVID-19 may lead to disease reactivation, with signs of hyperinflammation syndrome [83]. 

Moreover, other specific compounds (e.g., KRP-203, ponesimod and cenerimod, siponimod and ozanimod, and amiselimod and etrosimod) that seem to discriminate better between the various S1PR subtypes and retain greater efficacy and tolerability have been developed and are currently being studied [84]. New drugs modulating SL metabolism are currently under development or have been developed, and some have actually been studied as potential therapies for COVID-19 [85]. In particular, recent data suggest that opaganib, a selective inhibitor of SphK2 in oral pill form, reduces three key enzymes involved in SL metabolism: SphK2, DES, and GCS, while still retaining potent antiviral activity and the ability to target a range of other viruses [86]. Due to its dual activity, both anti-inflammatory and antiviral, this molecule has also been proposed for the treatment of patients hospitalized with COVID-19 as it can be safely administered, achieving a 62% reduction in mortality in a large sub-population of patients with moderately severe disease [86].

Fenretinide (4-HPR) is a synthetic retinoid compound, SPT agonist, and DES inhibitor, which has been studied in cancer and neurological diseases, as well as cardiometabolic (obesity and diabetes) and infectious (e.g., periodontitis by Aggregatibacter actinomycetemcomitans) scenarios; this is also due to its favorable toxicological profile [87,88,89,90]. Recent in vitro experiments showed that 4-HPR administration changes the SL profile and suppresses membrane fusion by decreasing membrane fluidity, which might be one of the factors triggering the inhibition of the SARS-CoV-2 infection [91]. Thus, taking into account its effect on SL metabolism and considering other additional beneficial properties (e.g., antitumoral, anti-inflammatory, antiviral, and immunomodulating characteristics), as well as the low-toxicity profile observed in clinical trials and long-term treatments, fenretinide could represent a potential promising adjuvant candidate drug in the treatment of COVID-19 [92,93].

Additionally, several antidepressants act as functional inhibitors of acid sphingomyelinase (FIASMAs): most of them are FDA-approved, characterized by low toxicity, and used for many therapeutic applications [94]. With regard to COVID-19, there is evidence of the use of FIASMAs as these antidepressants interfere with different steps of the SARS-CoV-2 life cycle by acting through the Cer system. Another possible target of drug therapy against COVID-19 concerns the interaction of SARS-CoV-2 with ACE2 receptors, which activates ASM, leading to the production and accumulation of Cer at the cell surface. Among antidepressant drugs, Amitriptyline reduces ASM levels and activity with a consequent reduction of Cer accumulation, decreasing SARS-CoV-2 uptake in human epithelial cells [3]. Moreover, antidepressants may also influence autophagy: accordingly, Fluoxetine has been shown to reduce SARS-CoV-2 replication by interfering with endolysosome acidification and cholesterol levels [19]. Through computational molecular docking and dynamic simulation approaches, identification of potential ASM inhibitors was performed; hence, Dutasteride, Cepharanthine, and Zafirlukast were recently identified with the lowest binding affinity scores (−9.7, −9.6, and −9.5 kcal/mol, respectively). Furthermore, computational ADME analysis highlighted the non-toxic properties of Cepharanthine and Zafirlukast, thus suggesting these inhibitors as potential promising tools for the inhibition of SARS-CoV-2 infection [95]. Indeed, these drugs have been shown to lower Cer concentration, hindering SARS-CoV-2 entry into cells and reducing hyperinflammation and pro-inflammatory cytokines (e.g., IL-6), which are considered risk factors for severe disease progression (such as hypertension, obesity, and thromboembolic complications), as well as the incidence of intubation and mortality in COVID-19 [96,97]. In particular, analysis of data obtained in in silico, in vitro, or in vivo studies suggested the use of 49 FIASMAs in the treatment of SARS-CoV-2 infection, although outcomes from randomized double-blind clinical trials are available only for amlodipine and fluvoxamine [98]. Moreover, recent findings also suggested antidepressants involved in SL-controlled autophagy (a cellular catabolic process leading to lysosomal degradation and recycling of proteins and organelles) linked to lysosomal proton pump (ATP-ase) inactivation and block of extracellular Cer-rich domain formation [99,100].

Other inhibitors targeting DES or SphK1 and -2 are being evaluated, mainly to assess their role against some types of cancer. Among these compounds, the following are currently under study:

- GT-11: this is a cyclopropene Cer molecule, where the C4–C5 double bond of Cer is replaced by a cyclopropene unit. GT-11 and its analogues act as DES1 inhibitors and have been studied mainly in cancer models, where they increase autophagy and apoptosis and inhibit tumor growth in mice models [101,102,103]. Interestingly, GT-11 is also able to inhibit flavivirus infection (e.g., West Nile virus) in a dose-dependent manner through DES inhibition [104].
- XM462: this molecule is a dihydroceramide analogue, where the C5 methylene group of the sphinganine moiety is replaced by a sulfur atom. XM462 and its analogues may inhibit both DES and ASAH1, although with different efficacy according to the characteristics of the N-acyl group [105]. Similar to another DES inhibitor (XM461), this compound can modulate autophagy and reduce amyloid secretion in primary neurons of a transgenic Alzheimer’s Disease model [106].
- SKI II: Sphingosine kinase inhibitor II, which interferes with SphK1 and SphK2, as well as DES1 [107], has been tested in the field of viral diseases, where it has been shown to reduce the virus replication of measles in both epithelial and lymphoid cell lines and affect mTOR Complex 1 (mTORC1) downstream signaling, likely through multiple mechanisms (e.g., reduction of rpS6 protein expression as well as its phosphorylation) [108,109];
- PF-543: this is a novel potent and selective inhibitor of SphK1 that has been studied in breast and colon cancers (IC50 = 2 nM; Ki = 3.6 nM: >100-fold selectivity for Sphk1 over Sphk2) [110].

## 7. Conclusions

The need for a deeper understanding of phospholipid’s role in infection grows parallel to increased knowledge of their role in virus pathogenic mechanisms, especially with regard to COVID-19. Emerging data demonstrate how these molecules can be useful in clinical practice to stratify disease severity and even guide clinical decisions following SARS-CoV-2 infection [111]. Interestingly, multiple pharmacological strategies can target SLs, some of which have already been explored in different clinical settings. However, knowledge about their potential antiviral properties and their related in vivo side effects (especially with regard to human applications) is still limited; this is mainly due to their recent availability. The same applies to other recently developed compounds, which have been investigated mainly through in vitro models [112]. Thus, future work might evaluate whether these molecules involved in the modulation of SL metabolism are also able to reduce viral replication in vivo and accelerate viral clearance, thereby retaining the potential to be developed into therapeutic drugs for use in SARS-CoV-2 infection.

Actually, given the intricate metabolic network of Cers, the future challenge will be to develop drugs targeting a specific small number of Cers while avoiding affecting other species, thus keeping and focusing on key cellular processes. However, to achieve this goal, the following considerations related to Cers have to be considered:

1. different acyl chain lengths not only characterize Cers structurally but also influence their physiological effects, which may be beneficial or detrimental depending on their molecular structure;
2. Cers characterized by the same acyl chain length may play different physiological functions and pathophysiological roles according to their localization in the cellular microenvironment;
3. it is still unknown whether the modulation of specific Cers may induce adverse changes in other species, which might be equally harmful [18,113];
4. Cers can interact with other drugs, as shown in the cancer field, and these interactions need to be carefully taken into account [114].

In any case, despite the complexity of the sphingolipid milieu and the possible interactions with other drugs, whose health implications are still unknown, the discovery, refinement, and application of molecules able to modulate sphingolipid metabolism with reliable and selective actions could pave the way for new supplementary pharmacological strategies and therapeutic tools in many clinical settings, including the viral context.

## Data Availability

Not applicable.

## Abbreviations

SARS-CoV-2	Severe acute respiratory syndrome coronavirus 2
COVID-19	Coronavirus disease 2019
COVID-ARDS	COVID-19—associated acute respiratory distress syndrome
Cer	Ceramides
SL	Sphingolipid
ASM	Acid sphingomyelinase
S1P	Sphingosine-1-phosphate
S1PR1	Sphingosine-1-phosphate receptor 1
INF-α	Interferon alpha
ORF1a; ORF1b	Open reading frames 1a and 1b
S	Spike protein
E	Envelope protein
M	Membrane protein
N	Nucleocapsid protein
ACE2	Angiotensin-converting enzyme 2
RDB	receptor-binding domain
NSPs	non-structural proteins
RTC	replication-transcription complex
RdRp	RNA-dependent RNA polymerase
ER	endoplasmic reticulum
ERGIC	ER-to-Golgi intermediate compartment
Sph	sphingosine
CerS	ceramide synthase
DHCer	dihydroceramide
GLS	glycosphingolipids
SM	sphingomyelins
SPT	serine palmitoyltransferase
DES1	dihydroceramide desaturase-1
CERT	Cer transport protein
SMS	sphingomyelin synthase
DAG	diacylglycerol
CerK	Cer kinase
C1P	Cer-1-phosphate
SMases	sphingomyelinases
CDases	ceramidases
ASAH1	acid CDase
ASAH2	neutral CDase
ACER 1-2-3	Alkaline CDases
SphK1-2	sphingosine kinases 1 and 2
HSV-1	Herpes simplex virus
GCS	glucosylceramide synthase
SERINC5	Serine Incorporator 5
RSV	resveratrol
mTORC1	mTOR Complex 1

## References

[1] World Health Organization Coronavirus Disease (COVID-19). Situation Reports. https://www.who.int/emergencies/diseases/novel-coronavirus-2019/situation-reports.

[2] Gaggini M., Pingitore A., Vassalle C. (2021). Plasma Ceramides Pathophysiology, Measurements, Challenges, and Opportunities. Metabolites.

[3] Carpinteiro A., Edwards M.J., Hoffmann M., Kochs G., Gripp B., Weigang S., Adams C., Carpinteiro E., Gulbins A., Keitsch S. (2020). Pharmacological Inhibition of Acid Sphingomyelinase Prevents Uptake of SARS-CoV-2 by Epithelial Cells. Cell Rep. Med..

[4] Carpinteiro A., Gripp B., Hoffmann M., Pöhlmann S., Hoertel N., Edwards M.J., Kamler M., Kornhuber J., Becker K.A., Gulbins E. (2021). Inhibition of acid sphingomyelinase by ambroxol prevents SARS-CoV-2 entry into epithelial cells. J. Biol. Chem..

[5] Vitner E.B., Achdout H., Avraham R., Politi B., Cherry L., Tamir H., Yahalom-Ronen Y., Paran N., Melamed S., Erez N. (2021). Glucosylceramide synthase inhibitors prevent replication of SARS-CoV-2 and influenza virus. J. Biol. Chem..

[6] Torretta E., Garziano M., Poliseno M., Capitanio D., Biasin M., Santantonio T.A., Clerici M., Lo Caputo S., Trabattoni D., Gelfi C. (2021). Severity of COVID-19 Patients Predicted by Serum Sphingolipids Signature. Int. J. Mol. Sci..

[7] Marfia G., Navone S., Guarnaccia L., Campanella R., Mondoni M., Locatelli M., Barassi A., Fontana L., Palumbo F., Garzia E. (2021). Decreased serum level of sphingosine-1-phosphate: A novel predictor of clinical severity in COVID-19. EMBO Mol. Med..

[8] Al-Kuraishy H.M., Batiha G.E., Al-Gareeb A.I., Al-Harcan N.A.H., Welson N.N. (2023). Receptor-dependent effects of sphingosine-1-phosphate (S1P) in COVID-19: The black side of the moon. Mol. Cell. Biochem..

[9] Pizzato M., Baraldi C., Boscato Sopetto G., Finozzi D., Gentile C., Gentile M.D., Marconi R., Paladino D., Raoss A., Riedmiller I. (2022). SARS-CoV-2 and the Host Cell: A Tale of Interactions. Front. Virol..

[10] V’kovski P., Kratzel A., Steiner S., Stalder H., Thiel V. (2021). Coronavirus biology and replication: Implications for SARS-CoV-2. Nat. Rev. Microbiol..

[11] Sternberg A., Naujokat C. (2020). Structural features of coronavirus SARS-CoV-2 spike protein: Targets for vaccination. Life Sci..

[12] Jackson C.B., Farzan M., Chen B., Choe H. (2022). Mechanisms of SARS-CoV-2 entry into cells. Nat. Rev. Mol. Cell Biol..

[13] Barua A., Grot N., Plawski A. (2022). The basis of mink susceptibility to SARS-CoV-2 infection. J. Appl. Genet..

[14] Eastman R.T., Roth J.S., Brimacombe K.R., Simeonov A., Shen M., Patnaik S., Hall M.D. (2020). Remdesivir: A Review of Its Discovery and Development Leading to Emergency Use Authorization for Treatment of COVID-19. ACS Cent. Sci..

[15] Sender R., Bar-On Y.M., Gleizer S., Bernshtein B., Flamholz A., Phillips R., Milo R. (2021). The total number and mass of SARS-CoV-2 virions. Proc. Natl. Acad. Sci. USA.

[16] Meacci E., Pierucci F., Garcia-Gil M. (2022). Skeletal Muscle and COVID-19: The Potential Involvement of Bioactive Sphingolipids. Biomedicines.

[17] Hannun Y.A., Obeid L.M. (2018). Sphingolipids and their metabolism in physiology and disease. Nat. Rev. Mol. Cell Biol..

[18] Gaggini M., Fenizia S., Vassalle C. (2023). Sphingolipid Levels and Signaling via Resveratrol and Antioxidant Actions in Cardiometabolic Risk and Disease. Antioxidants.

[19] Törnquist K., Asghar M.Y., Srinivasan V., Korhonen L., Lindholm D. (2021). Sphingolipids as Modulators of SARS-CoV-2 Infection. Front. Cell Dev. Biol..

[20] Futerman A.H., Hannun Y.A. (2004). The complex life of simple sphingolipids. EMBO Rep..

[21] Ryan E., Nguyen C.Q.N., Shiea C., Reid G.E. (2017). Detailed Structural Characterization of Sphingolipids via 193 nm Ultraviolet Photodissociation and Ultra High Resolution Tandem Mass Spectrometry. J. Am. Soc. Mass Spectrom..

[22] Gault C.R., Obeid L.M., Hannun Y.A. (2010). An overview of sphingolipid metabolism: From synthesis to breakdown. Adv. Exp. Med. Biol..

[23] Rechberger G.N., Wenk M.R. (2016). Sphingoid Bases. Encyclopedia of Lipidomics.

[24] Hirabayashi Y., Igarashi Y., Merrill A.H., Hirabayashi Y., Igarashi Y., Merrill A.H. (2006). Sphingolipids Synthesis, Transport and Cellular Signaling. Sphingolipid Biology.

[25] Sokolowska E., Blachnio-Zabielska A. (2019). The Role of Ceramides in Insulin Resistance. Front. Endocrinol..

[26] Kitatani K., Idkowiak-Baldys J., Hannun Y.A. (2008). The sphingolipid salvage pathway in ceramide metabolism and signaling. Cell. Signal..

[27] Kraveka J.M., Hannun Y.A., Lajtha A., Tettamanti G., Goracci G. (2009). Bioactive Sphingolipids: An Overview on Ceramide, Ceramide 1-Phosphate Dihydroceramide, Sphingosine, Sphingosine 1-Phosphate. Handbook of Neurochemistry and Molecular Neurobiology: Neural Lipids.

[28] Quinville B.M., Deschenes N.M., Ryckman A.E., Walia J.S. (2021). A Comprehensive Review: Sphingolipid Metabolism and Implications of Disruption in Sphingolipid Homeostasis. Int. J. Mol. Sci..

[29] Tan-Chen S., Guitton J., Bourron O., Le Stunff H., Hajduch E. (2020). Sphingolipid Metabolism and Signaling in Skeletal Muscle: From Physiology to Physiopathology. Front. Endocrinol..

[30] Ferreira N.S., Engelsby H., Neess D., Kelly S.L., Volpert G., Merrill A.H., Futerman A.H., Færgeman N.J. (2017). Regulation of very-long acyl chain ceramide synthesis by acyl-CoA-binding protein. J. Biol. Chem..

[31] Hanada K., Kumagai K., Yasuda S., Miura Y., Kawano M., Fukasawa M., Nishijima M. (2003). Molecular machinery for non-vesicular trafficking of ceramide. Nature.

[32] Huitema K., van den Dikkenberg J., Brouwers J.F., Holthuis J.C. (2004). Identification of a family of animal sphingomyelin synthases. EMBO J..

[33] Diaz Escarcega R., McCullough L.D., Tsvetkov A.S. (2021). The Functional Role of Sphingosine Kinase 2. Front. Mol. Biosci..

[34] Di Pietro P., Izzo C., Abate A.C., Iesu P., Rusciano M.R., Venturini E., Visco V., Sommella E., Ciccarelli M., Carrizzo A. (2023). The Dark Side of Sphingolipids: Searching for Potential Cardiovascular Biomarkers. Biomolecules.

[35] Hammerschmidt P., Brüning J.C. (2022). Contribution of specific ceramides to obesity-associated metabolic diseases. Cell. Mol. Life Sci..

[36] Yu Z., Peng Q., Huang Y. (2019). Potential therapeutic targets for atherosclerosis in sphingolipid metabolism. Clin. Sci..

[37] Shalaby Y.M., Al Aidaros A., Valappil A., Ali B.R., Akawi N. (2021). Role of Ceramides in the Molecular Pathogenesis and Potential Therapeutic Strategies of Cardiometabolic Diseases: What we Know so Far. Front. Cell Dev. Biol..

[38] Beckmann N., Becker K.A. (2021). Ceramide and Related Molecules in Viral Infections. Int. J. Mol. Sci..

[39] Teichgräber V., Ulrich M., Endlich N., Riethmüller J., Wilker B., De Oliveira-Munding C.C., van Heeckeren A.M., Barr M.L., von Kürthy G., Schmid K.W. (2008). Ceramide accumulation mediates inflammation, cell death and infection susceptibility in cystic fibrosis. Nat. Med..

[40] Oskeritzian C.A., Milstien S., Spiegel S. (2007). Sphingosine-1-phosphate in allergic responses, asthma and anaphylaxis. Pharmacol. Ther..

[41] Tringali C., Giussani P. (2022). Ceramide and Sphingosine-1-Phosphate in Neurodegenerative Disorders and Their Potential Involvement in Therapy. Int. J. Mol. Sci..

[42] Kornhuber J., Hoertel N., Gulbins E. (2022). The acid sphingomyelinase/ceramide system in COVID-19. Mol. Psychiatry.

[43] Lu Y., Liu D.X., Tam J.P. (2008). Lipid rafts are involved in SARS-CoV entry into Vero E6 cells. Biochem. Biophys. Res. Commun..

[44] Teissier E., Pécheur E.I. (2007). Lipids as modulators of membrane fusion mediated by viral fusion proteins. Eur. Biophys. J..

[45] Dissanayake T.K., Yan B., Ng A.C., Zhao H., Chan G., Yip C.C., Sze K.H., To K.K. (2021). Differential role of sphingomyelin in influenza virus, rhinovirus and SARS-CoV-2 infection of Calu-3 cells. J. Gen. Virol..

[46] Moolamalla S.T.R., Balasubramanian R., Chauhan R., Priyakumar U.D., Vinod P.K. (2021). Host metabolic reprogramming in response to SARS-CoV-2 infection: A systems biology approach. Microb. Pathog..

[47] Carreira A.C., Ventura A.E., Varela A.R., Silva L.C. (2015). Tackling the biophysical properties of sphingolipids to decipher their biological roles. Biol. Chem..

[48] Edwards M.J., Becker K.A., Gripp B., Hoffmann M., Keitsch S., Wilker B., Soddemann M., Gulbins A., Carpinteiro E., Patel S.H. (2020). Sphingosine prevents binding of SARS-CoV-2 spike to its cellular receptor ACE2. J. Biol. Chem..

[49] Vitner E.B., Avraham R., Politi B., Melamed S., Israely T. (2022). Elevation in sphingolipid upon SARS-CoV-2 infection: Possible implications for COVID-19 pathology. Life Sci. Alliance.

[50] Lang J., Bohn P., Bhat H., Jastrow H., Walkenfort B., Cansiz F., Fink J., Bauer M., Olszewski D., Ramos-Nascimento A. (2020). Acid ceramidase of macrophages traps herpes simplex virus in multivesicular bodies and protects from severe disease. Nat. Commun..

[51] Geiger N., Kersting L., Schlegel J., Stelz L., Fähr S., Diesendorf V., Roll V., Sostmann M., König E.M., Reinhard S. (2022). The Acid Ceramidase Is a SARS-CoV-2 Host Factor. Cells.

[52] Yan B., Yuan S., Cao J., Fung K., Lai P.M., Yin F., Sze K.H., Qin Z., Xie Y., Ye Z.W. (2022). Phosphatidic acid phosphatase 1 impairs SARS-CoV-2 replication by affecting the glycerophospholipid metabolism pathway. Int. J. Biol. Sci..

[53] Menck K., Sönmezer C., Worst T.S., Schulz M., Dihazi G.H., Streit F., Erdmann G., Kling S., Boutros M., Binder C. (2017). Neutral sphingomyelinases control extracellular vesicles budding from the plasma membrane. J. Extracell. Vesicles.

[54] Mesquita F.S., Abrami L., Sergeeva O., Turelli P., Qing E., Kunz B., Raclot C., Paz Montoya J., Abriata L.A., Gallagher T. (2021). S-acylation controls SARS-CoV-2 membrane lipid organization and enhances infectivity. Dev. Cell.

[55] Timilsina U., Umthong S., Ivey E.B., Waxman B., Stavrou S. (2022). SARS-CoV-2 ORF7a potently inhibits the antiviral effect of the host factor SERINC5. Nat. Commun..

[56] Göggel R., Winoto-Morbach S., Vielhaber G., Imai Y., Lindner K., Brade L., Brade H., Ehlers S., Slutsky A.S., Schütze S. (2004). PAF-mediated pulmonary edema: A new role for acid sphingomyelinase and ceramide. Nat. Med..

[57] Cartier A., Hla T. (2019). Sphingosine 1-phosphate: Lipid signaling in pathology and therapy. Science.

[58] Petrache I., Pujadas E., Ganju A., Serban K.A., Borowiec A., Babbs B., Bronova I.A., Egersdorf N., Hume P.S., Goel K. (2023). Marked elevations in lung and plasma ceramide in COVID-19 linked to microvascular injury. JCI Insight.

[59] Havulinna A.S., Sysi-Aho M., Hilvo M., Kauhanen D., Hurme R., Ekroos K., Salomaa V., Laaksonen R. (2016). Circulating Ceramides Predict Cardiovascular Outcomes in the Population-Based FINRISK 2002 Cohort. Arter. Thromb. Vasc. Biol..

[60] Anroedh S., Hilvo M., Akkerhuis K.M., Kauhanen D., Koistinen K., Oemrawsingh R., Serruys P., van Geuns R.J., Boersma E., Laaksonen R. (2018). Plasma concentrations of molecular lipid species predict long-term clinical outcome in coronary artery disease patients. J. Lipid Res..

[61] Michelucci E., Rocchiccioli S., Gaggini M., Ndreu R., Berti S., Vassalle C. (2022). Ceramides and Cardiovascular Risk Factors, Inflammatory Parameters and Left Ventricular Function in AMI Patients. Biomedicines.

[62] Marathe S., Schissel S.L., Yellin M.J., Beatini N., Mintzer R., Williams K.J., Tabas I. (1998). Human vascular endothelial cells are a rich and regulatable source of secretory sphingomyelinase. Implications for early atherogenesis and ceramide-mediated cell signaling. J. Biol. Chem..

[63] Schissel S.L., Keesler G.A., Schuchman E.H., Williams K.J., Tabas I. (1998). The cellular trafficking and zinc dependence of secretory and lysosomal sphingomyelinase, two products of the acid sphingomyelinase gene. J. Biol. Chem..

[64] Janneh A.H., Kassir M.F., Dwyer C.J., Chakraborty P., Pierce J.S., Flume P.A., Li H., Nadig S.N., Mehrotra S., Ogretmen B. (2021). Alterations of lipid metabolism provide serologic biomarkers for the detection of asymptomatic versus symptomatic COVID-19 patients. Sci. Rep..

[65] Song J.-W., Lam S.M., Fan X., Cao W.-J., Wang S.-Y., Tian H., Chua G.H., Zhang C., Meng F.-P., Xu Z. (2020). Omics-Driven Systems Interrogation of Metabolic Dysregulation in COVID-19 Pathogenesis. Cell Metab..

[66] Khodadoust M.M. (2021). Inferring a causal relationship between ceramide levels and COVID-19 respiratory distress. Sci. Rep..

[67] Ghidoni R., Caretti A., Signorelli P. (2015). Role of Sphingolipids in the Pathobiology of Lung Inflammation. Mediat. Inflamm..

[68] Ng T.W., Ooi E.M., Watts G.F., Chan D.C., Weir J.M., Meikle P.J., Barrett P.H. (2014). Dose-dependent effects of rosuvastatin on the plasma sphingolipidome and phospholipidome in the metabolic syndrome. J. Clin. Endocrinol. Metab..

[69] Huang K., Wen X.Q., Ren N., Yang L., Gao B. (2021). Lipidomic profile in patients with a very high risk of atherosclerotic cardiovascular disease on PCSK9 inhibitor therapy. Rev. Cardiovasc. Med..

[70] Palacios-Rápalo S.N., De Jesús-González L.A., Cordero-Rivera C.D., Farfan-Morales C.N., Osuna-Ramos J.F., Martínez-Mier G., Quistián-Galván J., Muñoz-Pérez A., Bernal-Dolores V., Del Ángel R.M. (2021). Cholesterol-Rich Lipid Rafts as Platforms for SARS-CoV-2 Entry. Front. Immunol..

[71] Sorice M., Misasi R., Riitano G., Manganelli V., Martellucci S., Longo A., Garofalo T., Mattei V. (2020). Targeting Lipid Rafts as a Strategy Against Coronavirus. Front. Cell Dev. Biol..

[72] Mazzone A., Clemente A., Chiappino D., Berti S., Vassalle C. (2018). Double Face of Statins at the Crossroad of Coronary Atherosclerotic Plaque and Aortic Valve Calcification?. JACC Cardiovasc. Imaging.

[73] Vahedian-Azimi A., Mohammadi S.M., Heidari Beni F., Banach M., Guest P.C., Jamialahmadi T., Sahebkar A. (2021). Improved COVID-19 ICU admission and mortality outcomes following treatment with statins: A systematic review and meta-analysis. Arch. Med. Sci..

[74] Lao U.S., Law C.F., Baptista-Hon D.T., Tomlinson B. (2022). Systematic Review and Meta-Analysis of Statin Use and Mortality, Intensive Care Unit Admission and Requirement for Mechanical Ventilation in COVID-19 Patients. J. Clin. Med..

[75] Ren Y., Wang G., Han D. (2023). Statins in hospitalized COVID-19 patients: A systematic review and meta-analysis of randomized controlled trials. J. Med. Virol..

[76] Vuorio A., Kovanen P.T. (2021). PCSK9 inhibitors for COVID-19: An opportunity to enhance the antiviral action of interferon in patients with hypercholesterolaemia. J. Intern. Med..

[77] Navarese E.P., Podhajski P., Gurbel P.A., Grzelakowska K., Ruscio E., Tantry U., Magielski P., Kubica A., Niezgoda P., Adamski P. (2023). PCSK9 Inhibition During the Inflammatory Stage of SARS-CoV-2 Infection. J. Am. Coll. Cardiol..

[78] Domi E., Hoxha M., Kolovani E., Tricarico D., Zappacosta B. (2022). The Importance of Nutraceuticals in COVID-19: What’s the Role of Resveratrol?. Molecules.

[79] Xiao Z., Ye Q., Duan X., Xiang T. (2021). Network Pharmacology Reveals That Resveratrol Can Alleviate COVID-19-Related Hyperinflammation. Dis. Markers.

[80] Nixon G.F. (2009). Sphingolipids in inflammation: Pathological implications and potential therapeutic targets. Br. J. Pharmacol..

[81] Chiba K. (2020). Discovery of fingolimod based on the chemical modification of a natural product from the fungus, Isaria sinclairii. J. Antibiot..

[82] Foerch C., Friedauer L., Bauer B., Wolf T., Adam E.H. (2020). Severe COVID-19 infection in a patient with multiple sclerosis treated with fingolimod. Mult. Scler. Relat. Disord..

[83] Gomez-Mayordomo V., Montero-Escribano P., Matías-Guiu J.A., González-García N., Porta-Etessam J., Matías-Guiu J. (2021). Clinical exacerbation of SARS-CoV2 infection after fingolimod withdrawal. J. Med. Virol..

[84] Huwiler A., Zangemeister-Wittke U. (2018). The sphingosine 1-phosphate receptor modulator fingolimod as a therapeutic agent: Recent findings and new perspectives. Pharmacol. Ther..

[85] McGowan E.M., Haddadi N., Nassif N.T., Lin Y. (2020). Targeting the SphK-S1P-SIPR Pathway as a Potential Therapeutic Approach for COVID-19. Int. J. Mol. Sci..

[86] Smith C.D., Maines L.W., Keller S.N., Katz Ben-Yair V., Fathi R., Plasse T.F., Levitt M.L. (2022). Recent Progress in the Development of Opaganib for the Treatment of Covid-19. Drug Des. Dev. Ther..

[87] Orienti I., Francescangeli F., De Angelis M.L., Fecchi K., Bongiorno-Borbone L., Signore M., Peschiaroli A., Boe A., Bruselles A., Costantino A. (2019). A new bioavailable fenretinide formulation with antiproliferative, antimetabolic, and cytotoxic effects on solid tumors. Cell Death Dis..

[88] Potenza R.L., Lodeserto P., Orienti I. (2022). Fenretinide in Cancer and Neurological Disease: A Two-Face Janus Molecule. Int. J. Mol. Sci..

[89] Thompson D., Mahmood S., Morrice N., Kamli-Salino S., Dekeryte R., Hoffmann P.A., Doherty M.K., Whitfield P.D., Delibegović M., Mody N. (2023). Fenretinide inhibits obesity and fatty liver disease but induces Smpd3 to increase serum ceramides and worsen atherosclerosis in LDLR^−/−^ mice. Sci. Rep..

[90] Yu H., Valerio M., Bielawski J. (2013). Fenretinide inhibited de novo ceramide synthesis and proinflammatory cytokines induced by Aggregatibacter actinomycetemcomitans. J. Lipid Res..

[91] Hayashi Y., Tsuchiya K., Yamamoto M., Nemoto-Sasaki Y., Tanigawa K., Hama K., Ueda Y., Tanikawa T., Gohda J., Maeda K. (2021). N-(4-Hydroxyphenyl) Retinamide Suppresses SARS-CoV-2 Spike Protein-Mediated Cell-Cell Fusion by a Dihydroceramide Δ4-Desaturase 1-Independent Mechanism. J. Virol..

[92] Orienti I., Gentilomi G.A., Farruggia G. (2020). Pulmonary Delivery of Fenretinide: A Possible Adjuvant Treatment In COVID-19. Int. J. Mol. Sci..

[93] Veronesi U., Mariani L., Decensi A., Formelli F., Camerini T., Miceli R., Di Mauro M.G., Costa A., Marubini E., Sporn M.B. (2006). Fifteen-year results of a randomized phase III trial of fenretinide to prevent second breast cancer. Ann. Oncol..

[94] Mir I.H., Thirunavukkarasu C. (2023). The relevance of acid sphingomyelinase as a potential target for therapeutic intervention in hepatic disorders: Current scenario and anticipated trends. Arch. Toxicol..

[95] Naz A., Asif S., Alwutayd K.M., Sarfaraz S., Abbasi S.W., Abbasi A., Alenazi A.M., Hasan M.E. (2023). Repurposing FIASMAs against Acid Sphingomyelinase for COVID-19: A Computational Molecular Docking and Dynamic Simulation Approach. Molecules.

[96] Hoertel N., Sánchez-Rico M., Gulbins E., Kornhuber J., Carpinteiro A., Lenze E.J., Reiersen A.M., Abellán M., de la Muela P., Vernet R. (2021). Association Between FIASMAs and Reduced Risk of Intubation or Death in Individuals Hospitalized for Severe COVID-19: An Observational Multicenter Study. Clin. Pharmacol. Ther..

[97] Darquennes G., Le Corre P., Le Moine O., Loas G. (2021). Association between Functional Inhibitors of Acid Sphingomyelinase (FIASMAs) and Reduced Risk of Death in COVID-19 Patients: A Retrospective Cohort Study. Pharmaceuticals.

[98] Loas G., Le Corre P. (2021). Update on Functional Inhibitors of Acid Sphingomyelinase (FIASMAs) in SARS-CoV-2 Infection. Pharmaceuticals.

[99] Gulbins A., Schumacher F., Becker K.A., Wilker B., Soddemann M., Boldrin F., Müller C.P., Edwards M.J., Goodman M., Caldwell C.C. (2018). Antidepressants act by inducing autophagy controlled by sphingomyelin-ceramide. Mol. Psychiatry.

[100] Pauletto P.J.T., Delgado C.P., da Rocha J.B.T. (2023). Acid sphingomyelinase (ASM) and COVID-19: A review of the potential use of ASM inhibitors against SARS-CoV-2. Cell Biochem. Funct..

[101] Bedia C., Triola G., Casas J., Llebaria A., Fabriàs G. (2005). Analogs of the dihydroceramide desaturase inhibitor GT11 modified at the amide function: Synthesis and biological activities. Org. Biomol. Chem..

[102] Holliday M.W., Cox S.B., Kang M.H., Maurer B.J. (2013). C22:0- and C24:0-dihydroceramides confer mixed cytotoxicity in T-cell acute lymphoblastic leukemia cell lines. PLoS ONE.

[103] Hernández-Tiedra S., Fabriàs G., Dávila D., Salanueva Í.J., Casas J., Montes L.R., Antón Z., García-Taboada E., Salazar-Roa M., Lorente M. (2016). Dihydroceramide accumulation mediates cytotoxic autophagy of cancer cells via autolysosome destabilization. Autophagy.

[104] Jiménez de Oya N., San-Félix A., Casasampere M., Blázquez A.B., Mingo-Casas P., Escribano-Romero E., Calvo-Pinilla E., Poderoso T., Casas J., Saiz J.C. (2023). Pharmacological Elevation of Cellular Dihydrosphingomyelin Provides a Novel Antiviral Strategy against West Nile Virus Infection. Antimicrob. Agents Chemother..

[105] Camacho L., Simbari F., Garrido M., Abad J.L., Casas J., Delgado A., Fabriàs G. (2012). 3-Deoxy-3,4-dehydro analogs of XM462. Preparation and activity on sphingolipid metabolism and cell fate. Bioorganic Med. Chem..

[106] Ordóñez-Gutiérrez L., Benito-Cuesta I., Abad J.L., Casas J., Fábrias G., Wandosell F. (2018). Dihydroceramide Desaturase 1 Inhibitors Reduce Amyloid-β Levels in Primary Neurons from an Alzheimer’s Disease Transgenic Model. Pharm. Res..

[107] French K.J., Schrecengost R.S., Lee B.D., Zhuang Y., Smith S.N., Eberly J.L., Yun J.K., Smith C.D. (2003). Discovery and evaluation of inhibitors of human sphingosine kinase. Cancer Res..

[108] Vijayan M., Seo Y.J., Pritzl C.J., Squires S.A., Alexander S., Hahm B. (2014). Sphingosine kinase 1 regulates measles virus replication. Virology.

[109] Chithelen J., Franke H., Länder N., Grafen A., Schneider-Schaulies J. (2022). The Sphingolipid Inhibitors Ceranib-2 and SKI-II Reduce Measles Virus Replication in Primary Human Lymphocytes: Effects on mTORC1 Downstream Signaling. Front. Physiol..

[110] Ju T., Gao D., Fang Z.Y. (2016). Targeting colorectal cancer cells by a novel sphingosine kinase 1 inhibitor PF-543. Biochem. Biophys. Res. Commun..

[111] Marín-Corral J., Rodríguez-Morató J., Gomez-Gomez A., Pascual-Guardia S., Muñoz-Bermúdez R., Salazar-Degracia A., Pérez-Terán P., Restrepo M.I., Khymenets O., Haro N. (2021). Metabolic Signatures Associated with Severity in Hospitalized COVID-19 Patients. Int. J. Mol. Sci..

[112] Pyne S., Adams D.R., Pyne N.J. (2016). Sphingosine 1-phosphate and sphingosine kinases in health and disease: Recent advances. Prog. Lipid Res..

[113] Hammerschmidt P., Ostkotte D., Nolte H., Gerl M.J., Jais A., Brunner H.L., Sprenger H.G., Awazawa M., Nicholls H.T., Turpin-Nolan S.M. (2019). CerS6-Derived Sphingolipids Interact with Mff and Promote Mitochondrial Fragmentation in Obesity. Cell.

[114] Shaw J., Costa-Pinheiro P., Patterson L., Drews K., Spiegel S., Kester M. (2018). Novel Sphingolipid-Based Cancer Therapeutics in the Personalized Medicine Era. Adv. Cancer Res..

